# Fecal microbiota transplantation for chronic constipation following bowel surgery in an elderly patient: A case report

**DOI:** 10.1097/MD.0000000000049600

**Published:** 2026-07-03

**Authors:** De-Xiang Liao, Shuai Zhang, Ding Cao, Xun Cai, Hui-Ling Sun, Wei-Dong Jin, Tao Fu

**Affiliations:** a School of Medicine, Wuhan University of Science and Technology, Wuhan, China; b Department of General Surgery, Central Theater Command General Hospital of PLA, Wuhan, China.

**Keywords:** case report, chronic constipation, fecal microbiota transplantation, intestinal dysbiosis

## Abstract

**Rationale:**

Chronic constipation is a common gastrointestinal disorder traditionally managed with laxatives and surgical interventions. Nevertheless, a subset of elderly patients remains unresponsive to conventional treatments. Additionally, chronic constipation is linked to an increased risk of mortality from cardiovascular events and a heightened incidence of colorectal cancer.

**Patient concerns:**

A 65-year-old male with a medical history of sigmoidostomy, enterostomy, and jejunostomy presented with an 11-year history of intermittent abdominal distension, pain, and constipation refractory to standard treatments including lactulose, castor oil, and glycerin enemas.

**Diagnoses:**

The patient was diagnosed with severe chronic constipation associated with surgically altered anatomy and intestinal dysbiosis.

**Interventions:**

After thorough discussion, fecal microbiota transplantation (FMT) was implemented as a therapeutic intervention.

**Outcomes:**

The patient’s abdominal distension and pain were significantly alleviated within 1 week and completely resolved 6 months post-FMT. No recurrence was observed during the subsequent follow-up period.

**Lessons:**

This case demonstrates that FMT can serve as an effective therapeutic intervention for elderly patients with surgically altered anatomy and intestinal dysbiosis, particularly those showing reduced response to conventional laxative treatments for chronic constipation.

## 1. Introduction

Chronic constipation is a widespread gastrointestinal disorder, with a global prevalence exceeding 15%.^[1,2]^ It is primarily categorized into functional and organic constipation, with functional constipation being the more common type.^[3]^ The pathogenesis of chronic constipation is largely attributed to factors such as dysbiosis of the gut microbiota, dyssynergia of the pelvic floor muscles, and dysfunction of the enteric nervous system.^[1]^ Recent evidence increasingly suggests that chronic constipation is often associated with an imbalance in intestinal microbiota.^[4,5]^ Specifically, there is a significant reduction in the abundance of beneficial bacteria, including *Bacteroides*, *Bifidobacterium*, and *Lactobacillus*, in comparison to healthy individuals, while potential pathogenic bacteria such as *Escherichia coli*, *Staphylococcus aureus*, and Enterobacteriaceae are markedly increased.^[6]^

The global incidence of constipation has been increasing, influenced by factors such as lifestyle and dietary changes, psychosocial influences, and the aging population.^[7]^ Additionally, constipation is linked to heightened mortality risks from conditions like hypertension, myocardial infarction, and stroke, as well as an increased prevalence of colorectal cancer.^[8]^ Therefore, the prevention and management of chronic constipation, especially in elderly patients, are of critical importance.

Traditional management strategies for chronic constipation typically involve the use of laxatives and surgical interventions; however, these methods often have limited therapeutic efficacy due to the relatively high incidence of adverse effects associated with pharmacological treatments and the significant physical trauma resulting from surgical procedures. Fecal microbiota transplantation (FMT) has emerged as a promising microecological therapeutic approach, involving the transfer of gut microbiota from healthy donors to patients’ intestines to reconstruct the microbial community and alleviate chronic constipation.^[9]^ At present, there is a paucity of research regarding the application of FMT for the treatment of chronic constipation, especially in patients who exhibit symptoms subsequent to intestinal surgery. In light of this, we present a case report involving an elderly patient who developed chronic constipation over a decade following intestinal surgery, with notable symptom alleviation observed subsequent to FMT treatment.

## 2. Case presentation

We present the case of a 65-year-old male patient who exhibited intermittent abdominal distension and pain, accompanied by defecation difficulties, persisting for 11 years. The patient underwent a sigmoidostomy on August 5, 2014, due to rectal perforation and subsequently developed an abdominal infection on postoperative day 3, necessitating enterostomy and jejunostomy. Over the ensuing 11 years, the patient experienced recurrent episodes of abdominal distension and pain, predominantly localized to the mid to lower abdomen. The pain was described as a sensation of fullness that progressively worsened over time and was not alleviated by rest or changes in body position. Following these episodes, the patient encountered difficulty in passing gas and stool through the small intestinal stoma, often excreting hard, pellet-like feces. The Wexner constipation score (WCS) was recorded at 15, the Patient Assessment of Constipation Quality of Life (PAC-QOL) questionnaire score was 72, and the Bristol Stool Form Scale (BSFS) consistently indicated Type 1 stools. An abdominal X-ray revealed significant fecal accumulation throughout the intestinal tract, which contributed to the symptoms of distension and pain. Then the patient underwent a barium enema examination, which revealed markedly slowed colonic peristalsis, providing objective radiographic evidence of impaired colonic motility consistent with chronic constipation. Although treatments like lactulose, castor oil, and glycerin enemas initially worked, their effectiveness decreased, and abdominal pain persisted. After thorough discussions with the patient and family, FMT was started as a treatment.

After obtaining written informed consent from the patient, an analysis of the gut microbiota was performed, revealing a dysbiotic state that indicated suitability for FMT.

The specific detection methodology is as follows. Total genomic DNA was extracted from fecal samples using a commercial stool DNA extraction kit according to the manufacturer’s instructions. The V3–V4 hypervariable region of the bacterial 16S ribosomal DNA was amplified by polymerase chain reaction using universal primers. The amplified products were then subjected to high-throughput sequencing on a second-generation sequencing platform (Illumina MiSeq). Raw sequencing reads were quality-filtered and assembled. Clean reads were subsequently processed through either clustering into operational taxonomic units at 97% similarity or denoising to generate amplicon sequence variants. Taxonomic annotation was performed by alignment against the SILVA reference database, and microbial abundance profiles were generated to reveal the overall community composition.

Comprehensive preoperative evaluations were conducted to exclude any contraindications to FMT. FMT was performed using donor feces from a rigorously screened healthy donor. Donor screening was conducted in strict accordance with the Nanjing Consensus guidelines, including a detailed questionnaire to exclude high-risk behaviors, and negative serological tests for human immunodeficiency virus, hepatitis B virus, hepatitis C virus, and syphilis, as well as negative stool tests for common enteric pathogens and *Clostridioides difficile*.^[10]^ To prepare the fecal microbiota suspension, approximately 50 g of donor feces were homogenized with 200 mL of sterile normal saline using an automated microfiltration system, yielding a final bacterial load of approximately 1 × 10^8^ colony-forming units (CFU)/mL. The suspension was then cryopreserved at −80°C and thawed immediately prior to administration. A nasojejunal tube was inserted for microbial delivery. The thawed suspension was administered in two divided 200-mL aliquots via the nasojejunal tube on June 10 and June 12, respectively. No adverse events were observed during or after the infusion procedure. Following FMT, the patient reported significant relief from abdominal distension and pain, as well as improved stool passage. At the 6-month follow-up, symptomatic improvement was assessed, and the WCS, PAC-QOL score, and BSFS were documented (Table 1). Furthermore, a repeat analysis of gut microbiota composition was conducted 1-month post-FMT.

**Table 1:** Clinical scale scores used to assess the clinical efficacy of enterobacterial transplantation.

Project	Before treatment	After treatment	1-Month follow up	2-Month follow up	3-Month follow up	6-Month follow up
WCS	15	10	7	8	6	4
PAC-QOL	72	55	42	36	32	25
BSFS	1	2	3	3	4	4

BSFS = Bristol Stool Form Scale, PAC-QOL = patient assessment constipation quality-of-life questionnaire score, WCS = The Wexner constipation score.

**Figure 1.**
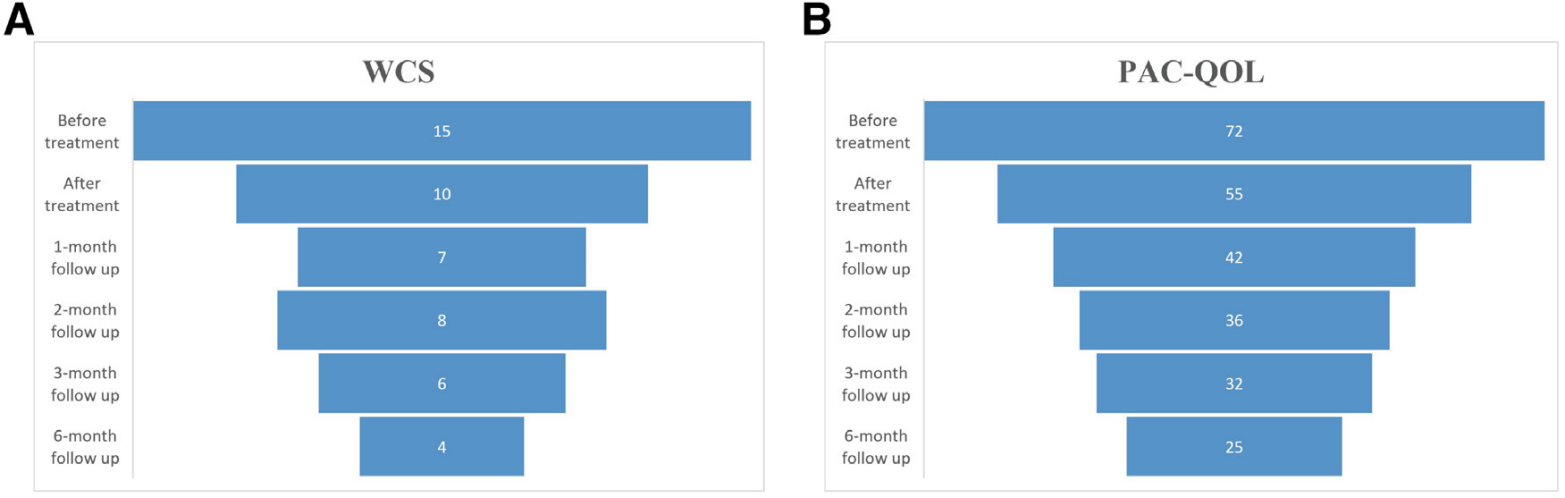
The efficacy of fecal microbiota transplantation (FMT) was comprehensively evaluated using the following clinical assessment tools, and the results are presented in funnel plots: (A) the Wexner constipation score (WCS) and (B) Patient Assessment Constipation Quality-of-Life Questionnaire (PAC-QOL).

**Figure 2.**
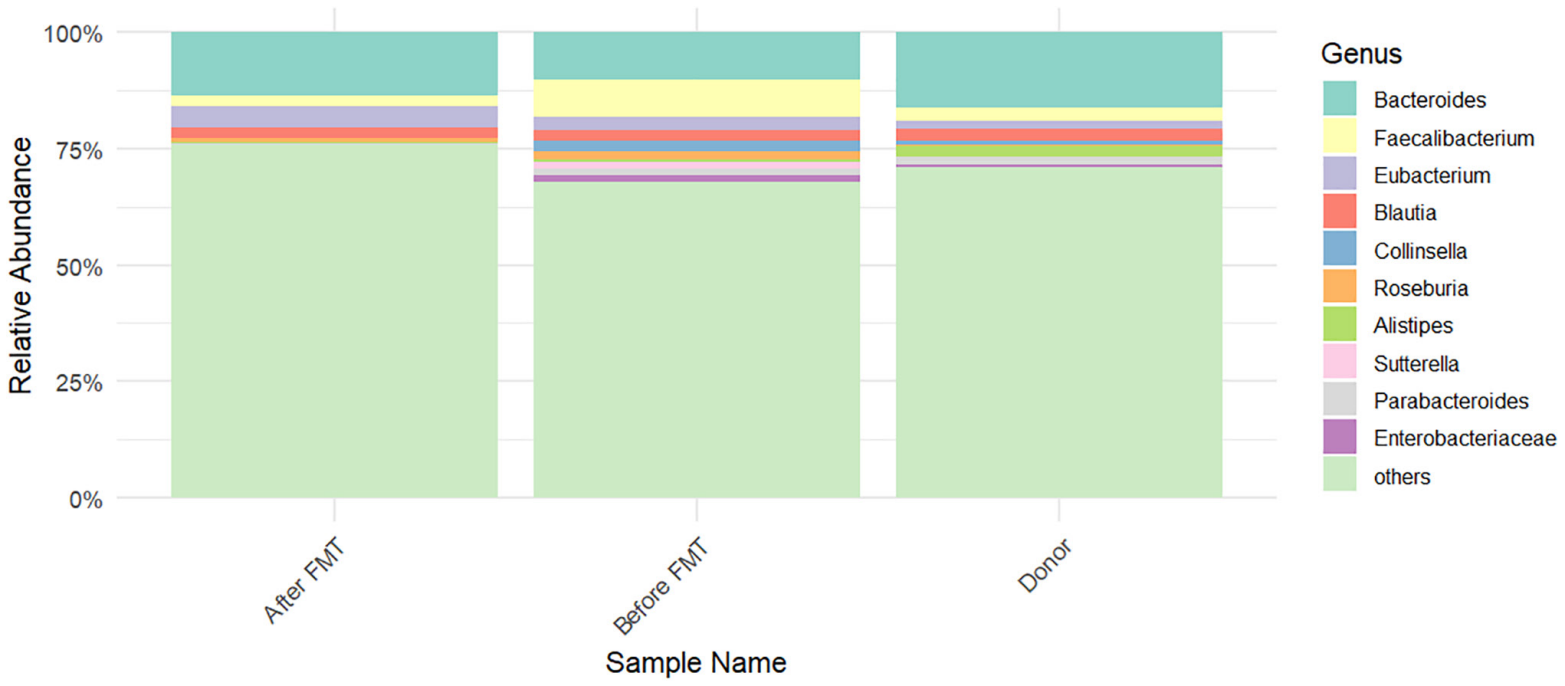
Fecal microbiota composition at the genus level in the patient with chronic functional constipation before and after treatment and in a healthy donor.

During the follow-up period, significant alleviation of abdominal distension and pain was observed 1 week following FMT. Abdominal discomfort was completely resolved 3-month posttreatment, with no recurrence detected in subsequent follow-ups. The WCSs began to decrease immediately after FMT, exhibited a marked reduction by the 1-month mark, and continued to decline gradually before stabilizing (Fig. 1A). The BSFS showed improvement towards normal within 1 week and achieved complete normalization after 3 months. The PAC-QOL score indicated a substantial reduction in constipation severity and a decreased impact on daily life (Fig. 1B), an effect that persisted throughout the follow-up period. Concurrently, analysis of gut microbiota composition revealed a notable increase in the abundance of *Bacteroides*, while *Sutterella* and *Faecalibacterium* were significantly reduced posttreatment (Fig. 2).

## 3. Discussion

This case illustrates that FMT may provide relief for chronic refractory constipation in elderly patients with a history of intestinal surgery. Gastrointestinal surgery can alter intestinal anatomy and disrupt the gut microbiota, potentially impairing intestinal motility. These effects may be further exacerbated in elderly individuals due to factors such as reduced dietary fiber intake, physical inactivity, a decrease in interstitial cells of Cajal, loss of cholinergic neurons within the intestinal musculature, and diminished smooth muscle function.^[7]^ In the present case, the patient exhibited several of these predisposing factors, including advanced age, prior abdominal infection, small bowel resection, colostomy, and a history of antibiotic use, all of which may have contributed to the development of intractable constipation.

Conventional pharmacological treatments for chronic constipation typically include bulk-forming, osmotic, and stimulant laxatives.^[1]^ These agents facilitate bowel movements by increasing stool water content and volume or by stimulating the enteric nervous system to enhance peristalsis and secretion. However, prolonged use of these treatments may result in complications, including intestinal obstruction and impaired absorption of calcium and iron. In the current case, the patient exhibited a progressive tolerance to laxatives, accompanied by increasing abdominal distension and pain, which ultimately necessitated the consideration of FMT. Recent research suggests that FMT for chronic constipation achieves remission rates exceeding 50% and improvement rates surpassing 60%,^[11]^ while presenting a lower incidence of adverse events compared to traditional treatments such as laxatives or surgical interventions. The therapeutic mechanisms underlying FMT may involve the correction of microbial dysbiosis, an increase in the abundance of butyrate-producing bacteria, and a reduction in methanogenic archaea, thereby alleviating methane-induced inhibition of intestinal smooth muscle contraction.^[9]^ Furthermore, microbial-derived short-chain fatty acids have the potential to stimulate enterochromaffin cells to synthesize serotonin, which enhances motility and supports the function of enteric glial cells. Additionally, certain gut microbes may influence intestinal motility through vagal nerve signaling and the gut–brain axis.^[12]^

In this case, pre-FMT gut microbiota analysis revealed a notable reduction in the abundance of *Bacteroides*, accompanied by an increase in *Sutterella* and *Faecalibacterium*. Previous studies have associated reduced *Bacteroides* with constipation, and elevated *Sutterella* has been linked to conditions such as autism spectrum disorder, irritable bowel syndrome, and inflammatory bowel disease. Following FMT, a marked decrease in *Sutterella* and *Faecalibacterium* was observed, alongside gradual alleviation of chronic constipation symptoms. While the decrease in *Sutterella* is consistent with its potential pathogenic role, the post-FMT reduction in *Faecalibacterium* – a genus generally considered beneficial due to its butyrate-producing and anti-inflammatory properties – is unexpected and may appear paradoxical. It is possible that the pre-FMT expansion of *Faecalibacterium* represented a compensatory response to dysbiosis, or that its relative abundance was influenced by cross-feeding interactions within an altered microbial network, rather than indicating a direct pathogenic role. Therefore, although these microbial shifts were temporally associated with clinical improvement, any causal relationship between specific taxa and chronic constipation remains speculative, and the observed changes may also reflect overall ecosystem remodeling rather than a simple increase or decrease of individual bacteria. Consequently, we refrain from drawing definitive conclusions about the roles of *Sutterella* and *Faecalibacterium* in constipation based on this single case, and emphasize that further mechanistic studies are required to clarify these associations.

For patients with risk profiles similar to the present case, early vigilance and timely pharmacological intervention for constipation are advisable. When tolerance to standard drug therapy develops, prompt gut microbiota analysis may help evaluate the suitability of FMT, with the aim of enhancing quality of life and reducing adverse events.

In summary, FMT exhibited promising results in this case. Nevertheless, several limitations must be acknowledged. First, clinical trials examining FMT for chronic constipation remain scarce, and the long-term efficacy and safety of this intervention have yet to be comprehensively established. Second, although FMT offers a novel therapeutic approach, the precise mechanisms by which the transplanted microbiota influence the pathophysiology of chronic constipation are not yet fully understood and warrant further investigation. The successful implementation of FMT in this elderly patient with postsurgical chronic constipation refractory to conventional therapy provides valuable clinical insights and underscores the need for future prospective studies to explore the potential of FMT in managing related intestinal disorders.

## 4. Conclusion

This case demonstrates that FMT can serve as an effective therapeutic intervention for elderly patients with surgically altered anatomy and intestinal dysbiosis, particularly those showing reduced response to conventional laxative treatments for chronic constipation. Although our study provides initial evidence, it is important to note that these results require confirmation in larger, prospective cohorts.

## Author contributions

**Conceptualization:** Shuai Zhang, Ding Cao.

**Supervision:** Xun Cai, Hui-Ling Sun, Wei-Dong Jin.

**Visualization:** De-Xiang Liao, Shuai Zhang, Ding Cao, Tao Fu.

**Writing – original draft:** De-Xiang Liao, Tao Fu.

**Writing – review & editing:** Xun Cai, Hui-Ling Sun, Wei-Dong Jin.
